# Well-Being Lessons for Improving Charities’ Online Recruitment

**DOI:** 10.3389/fpsyg.2019.02582

**Published:** 2019-11-22

**Authors:** Maria Buenadicha-Mateos, Maria Isabel Sánchez Hernández, Oscar R. González-López, Juan Luis Tato-Jiménez

**Affiliations:** Department of Business Organization and Sociology, School of Economics and Business Administration, University of Extremadura, Badajoz, Spain

**Keywords:** charities, future of work, non-profit organizations, online recruitment, voluntary sector

## Abstract

In an increasingly competitive context, attracting and retaining the best employees are a real preoccupation and a big challenge for organizations. Online recruitment (OR) is a growing trend, and corporate websites are an important instrument for talent attraction, but academic research on this topic is still scarce, especially in the voluntary sector. To shed light on the topic, this study examines and compares the 100 best companies to work for, published by Fortune, and the 100 largest charities, reported by Forbes. The comparative study focuses the attention and quantifies the web section devoted to careers, concretely information related to goods practices affecting the workers well-being. The results indicated, as essential in the OR process of charities, to understand the relevance of their web content because that affects the intentions of potential applicants. The work concludes that benchmarking efforts can be helpful for increasing the charities’ attractiveness in the labor market in the near future.

## Introduction

Nowadays, it is very well known that the future of work is a general hot topic in the socioeconomic arena (Balliester and Elsheikhi, 2018) that is being shaped by, at least, two powerful forces: digitalization and the role of talent (Manpower Group, 2017). On the one hand, the adoption of artificial intelligence in the workplace is changing the work force relations. This issue is still relatively misunderstood (Mital and Pennathur, 2004) and, probably because of that, a bit scary (McClure, 2018; Wolnicki and Piasecki, 2019). However, and related to human resources management (HRM), the positive role of technology has also been highlighted in, for instance, improving recruitment processes, and helping correct skills mismatches (Manyika et al., 2015). On the other hand, and looking for scale effects, the growing global competition is generating the integration of societies and economies. This current trend derives on the expansion of work and a widening wage inequality (Frey and Osborne, 2017) intensifying the competition for talent (Cappelli, 2000; McCauley and Wakefield, 2006; Pfeffer, 2010; Docquier and Machado, 2016).

In this context, and around the world, it has been observed that the voluntary sector is growing because it is increasingly offering services previously provided by the public sector (Defourny, 2001; Nickson et al., 2008). Considering that the quality of the services provided partially depends on their staff, the HRM is progressively claimed to play a determinant role in enhancing the efficiency of the sector (Ridder et al., 2012).

Few people would question that recruitment is a key strategic domain in HRM that add value to organizations. At the moment, online recruitment (OR) is a growing trend, specially designed for Millennials that will make up over a third of the global workforce by 2020 (ManpowerGroup, 2016), but academic research on this topic is still scarce.

Talent management, defined as the process through which organizations meet their needs for talent in strategic positions (Cappelli and Keller, 2014), is a big challenge for organizations in the twenty-first century (Ashton and Morton, 2005). Both recruitment in general and OR in particular have become a key factor in the so-called war for talent (Bostjancic and Slana, 2018). In addition, when planning a firm’s talent attraction strategy, the primary intent must be to become an employer of choice, and a great place to work at (Pandita and Ray, 2018).

The voluntary sector, also denoted as not-for-profit sector, third sector, or social economy, is constituted by different kinds of organizations that are not public and do not fall into the state or market categories (Corry, 2010), such as non-governmental organizations, charities, self-help groups, social enterprises, networks, or clubs, among others. Reviewing previous studies, academic literature on the topic suggests that HRM in the voluntary sector has traditionally lacked a refined approach (Lloyd, 1993; Cunningham, 1999; Kellock Hay et al., 2001; Parry et al., 2005; Akingbola, 2006; Ridder and McCandless, 2010). In addition, a number of difficulties exist for recruitment in the voluntary sector because of inadequate career progression, precarious job security, or poor wages (Cunningham, 2005).

However, in an increasingly competitive context, attracting and retaining the best employees are a real preoccupation and a big challenge for the voluntary sector (Nickson et al., 2008; Ariza-Montes et al., 2017). Recognizing that recruitment is a challenge for the sector, the research question is, what should be learned from the for-profit sector? To answer the question, the study focused on corporate webs as crucial contemporary means for attracting and retaining talent. In line with Keeman et al. (2017), given that well-being is important for organizational success, we put the attention on information related to the workers well-being. Following Giorgi et al. (2016), employee well-being is considered an umbrella concept including various services and benefits offered to employees with the aim of fostering their working conditions and professional growth such as interventions regarding allowances, housing, transportation, medical insurance, or wellness coverage (Schmitz and Schrader, 2015).

The purpose was to examine what practices coming up from the for-profit sector could be also applied in recruitment in the voluntary sector. The objective was to compare what kind of information disclosure related to workers well-being is provided in corporate webs in both sectors, with a sample conformed by the best organizations in each sector, as will be exposed later.

Although there is a lack of consensus on what exactly conforms the voluntary sector, Blackmore (2004) referred to non-profit organizations with an independent governance structure and set up for promoting a shared interest. In line with Parry et al. (2005), in this research, we focus on organizations involved with charity activities, such as education, relief of the poor, the advancement of religion, and other services considered to benefit the community. Our comparative study refines the understanding of the role of well-being practices in attracting new talent in both lucrative and voluntary sector. Our research also has implications for practitioners because the results may convince human resources (HR) managers to improve their recruitment strategies by reinforcing the emphasis on well-being in webpages to attract the new generations entering the labor market.

## Theoretical Framework

### Online Recruitment

The online recruitment, also known as e-recruitment, is the practice whereby web-based technology is used as a means of attracting and hiring personnel (Ghazzawi and Accoumeh, 2014). Recently, OR has been defined as “the use of communication technologies such as websites and social media to find and attract potential job applicants, to keep them interested in the organization during the selection processes, and to influence their job choice decisions” (Chapman and Gödöllei, 2017, p. 213).

Despite the fact that OR is still being considered a relatively new process in HRM, it is in fact an important source of recruitment (Ouirdi et al., 2016; Rosoiu and Popescu, 2016), and the number of jobseekers using this recruitment method is increasing (Petre et al., 2016).

Academic literature associates OR with both economic (e.g., cost reduction) and human resource system advantages (e.g., applicants’ resumes can be stored and organized in digital databases) (Din et al., 2015; Vidros et al., 2016). Another important advantage is that it could also improve the image that the organization communicates to candidates using systems that provide automatic feedback (Galanaki, 2002). In the same way, we can enumerate some disadvantages that the literature points out, like the risk of receiving a large number of unsuitable applicants or a higher number of applications that the organization could be not technologically prepared to manage (Barber, 2006). Finally, it is important to regard the amount of investment and its financial cost to both implement and manage an efficient technological system capable to complete the required OR process (Petre et al., 2016).

There has been little investigation about whether OR methods are better suited for particular types of organizations. In addition, corporate websites are an important instrument for OR, which requires specific studies. However, there is also little understanding of the properties and effectiveness of this technology, especially in the voluntary sector. This work is therefore intended to make contributions to the scarce literature on the topic.

### The Role of Web Pages

The corporate website is currently one of the cornerstones of firms’ communication strategies, being the fundamental instrument for information dissemination. Through corporate websites, organizations spread information related to the organization itself and also related to its products and services, as an important communication outlet for brands. Corporate websites have emerged as essential channels for distributing information to stakeholders (customers, suppliers, investors, partners, and employees among others) (Iaia et al., 2019). Simultaneously, stakeholders can also use new media for gathering information from the organization and build fruitful relationships with them (Sriramesh et al., 2013). Taking into account the complexity of stakeholders for a charity, the right development and administration of corporates websites have revealed as particularly important for these organizations (Goatman and Lewis, 2007).

Broadly, voluntary organizations have classically been considered as early adopters of new technology (Hackler and Saxton, 2007). For instance, Barnes and Mattson (2010) showed that non-profit organizations were more active than for profits in their use of social media tools such as blogs, podcasts, or Twitter. On the contrary, and related to HRM, when comparing small business and non-profit organizations, Witzig et al. (2012) demonstrated that small businesses appear to have greater adoption and usage rates of the professional networking site LinkedIn.

Voluntary organizations can use their websites to improve their visibility to the community, crowdsource info to resolve problems or collect votes to enlighten project priorities, fundraise, crowdfund their actions, and recruitment (Kirk et al., 2016). The importance of websites as a communication tool is even more critical in non-profits than in for-profit organizations because the task of engaging external stakeholders warrants non-profits’ ongoing existence (Hoefer and Twis, 2018). As the Internet continues to grow, voluntary organizations are starting to build and develop better relations with their stakeholders, and they are also changing how they communicate, basically thinking that message dissemination is no longer sufficient (Greenberg and MacAulay, 2009). However, and even with the influence of social media, the non-profits’ audience still uses their websites (McMahon et al., 2015; Kirk and Abrahams, 2017).

Considering the above, charities must reinforce their websites for attracting talent as part of their OR strategies. In this context, and according to Collings and Mellahi (2009), organizations must follow three steps to have an employee attraction strategy: employer branding, becoming an employer of choice, and creating a recognized great place to work. With these steps, the talent attraction strategy will be able to be successful (Swailes, 2016). In this context, physical and psychological well-being in the workplace should be a good point to make a company more appealing, and corporate websites should be a good means to show a healthy work environment, conducive to learning, performing, and socializing (Edwards, 2009; Pandita and Ray, 2018).

### Physical and Psychological Well-Being in the Workplace

According to a Positive Psychology approach, the well-being of people, groups, and organizations must be promoted (Seligman and Csikszentmihalyi, 2000; Henry, 2005; Di Fabio, 2017). Wellness in the workplace has been defined as “the integration of many dimensions, including emotional, intellectual, physical, spiritual, and social, that expands one’s potential to live and work effectively and to make a significant contribution to society” (Corbin and Lindsey, 1994, p.233). Employers have an important role in providing workplace practices that protect employee’s physical and psychological well-being.

An effective workplace strategy must simultaneously address the social, physical, and technical components of the work environment (Ellison Schriefer, 2005). Even acknowledging the wide diversity of compensations that organizations can offer to their workers, such as coverage of different medical assistance services for them and their families, for instance, these types of benefits are more related to insurance policies of health than to the promotion tools of well-being at work. Good practices in comprehensive workplace wellness programs generally include supportive physical and psychological environments.

Given the relative scarce knowledge on the topic, and array of potential benefits of wellness programs, it is necessary to approach and to investigate these programs further and deeper (Sabharwal et al., 2019). For instance, the workplace can provide an environment of social support with opportunities for direct communication with employees to support and encourage healthy lifestyle choices. Organizations have the possibility to develop wellness programs, consisting of employee fitness and massages activities providing assistance to employees with expenses. The aim is improving and maintaining employees’ state of physical fitness (Ryan et al., 2019). According to Madsen (2003), this kind of programs are an example of Education and Lifestyle Programs (ELP) within the wellness programs. In addition, the cafeteria programs (CP) can be a very suitable tool for healthy eating, wellness, and the promotion of social relationships at work (Wanjek, 2005; Dawson et al., 2006). WCP means that the company is providing and, partially or totally, supporting a cafeteria service (beverages and/or food).

Another important aspect related to work well-being is the satisfaction with the work commute. Olsson et al. (2013) demonstrated that it has a substantial influence on the general happiness of employees. Long work journeys in congested car traffic jams cause residual stress in the workplace (Novaco et al., 1990) and are associated with negative feelings during the workday (Kahneman et al., 2004). The existence of employee transport programs (ETP) means that firms offer transport benefits such as company cars, pass on public transportation, travel, and parking benefits to employees. It should be considered that the fact that the organization offers the company’s cars or travel and parking benefits generally makes the employees less sensitive to the real costs of daily commuting. In addition, transport benefits can discourage employees to move their residence because the employees’ expenses on commuting are reduced (Van Ommeren et al., 2006).

Moving to more intrinsic motivators related to well-being in the workplace, according to Madsen (2003), employee assistance programs (EAP) would be a good example. EAPs are a job-based programs operating within a work organization with the purposes of identifying troubled employees. These programs serve to motivate employees to solve their troubles and provide access to counseling or treatment for those who need these services (Sonnenstuhl and Trice, 2018). These programs assist employees with behavioral health issues, personal concerns, and work-related problems to change of behavior. The study of Nunes et al. (2018) provides empirical evidence that users of EAP tend to reduce their absenteeism at a faster pace than non-EAP users experiencing similar challenges to maintain productivity.

In the same vain, the employee recognition programs (ERP) are a way to acknowledge an employee’s outstanding performance and also motivate improvements (Luthans, 2000). These programs, aimed at reinforcing courageous behavior at work (Ali and Ahmed, 2009), use rewards to acknowledge employees for special contributions or exceptional efforts above the expectations stated in an individual’s job description (Hager et al., 2017). Beyond the effect on a specific individual, these programs can potentially provide a motivational effect beyond individual beneficiaries (Li et al., 2016). It is expected that the formal recognition of a team member will not only lead to positive changes in the individual but also to the collective performance of teammates.

Finally, and also related to intrinsic motivation and well-being, and directly connected to recruitment, the modern employee referral programs (ERFP) are remarkable. They serve to value employees who have suggested jobseekers to cover openings in the company. It is in fact an internal recruitment method, considered as an informal source (Breaugh and Starke, 2000), and employed by organizations to identify potential candidates from their existing employees’ social networks (Van Hoye, 2013). An ERFP encourages a company’s existing employees to select and recruit the suitable candidates from their social networks (Powell, 2009). ERFP involves more than bringing new employees into the workplace. It brings in particular coworkers, individuals known by insiders and likely to have strong social ties (Granovetter, 1973). Friebel et al. (2019) showed that ERFPs can have substantial benefits beyond generating referrals. The most supported mechanism is that workers value being involved in hiring. It is also important to highlight that there is a relationship among ERFP, rotation, and performance (Pieper et al., 2019).

## Research Design

### Sample

This study used the last Forbes’ “The Largest 100 U.S. Charities” list. It is a very well-known compendium of the top charitable organizations that Forbes publishes annually based on the amount of private support received by charities in the latest fiscal reporting period (Forbes, 2019). The list of for-profit organizations was gleaned from “The Fortune 100,” the 100 best companies to work for, a list of companies that are ranked by Fortune magazine (Fortune, 2019). It is remarkable that other academic works have previously considered the same or similar rankings (Moore et al., 2002; Bernardi et al., 2006; Witzig et al., 2012).

### Method and Procedure

There are some studies, in different contexts, that point out several website elements to attract the attention of the jobseekers such as usability, design, innovation, or the content of the information (Selden and Orenstein, 2011; Ehrhart et al., 2012; Allen D.G. et al., 2013). According to the signaling theory (Spence, 1973), the information provided on websites about the job and the organization may be used as informative signals by prospective applicants in determining their fit and attraction to the position (Gregory et al., 2013). The most basic requirement of successful OR is the information design and content provided by employers. This is an important matter because the information given by employers would influence job applicants in the initial stage of job application (Moghaddam et al., 2015), so that OR would help to assess and fit the best job applicants in organizations due to the fact that jobseekers are able to gather more and relevant information about companies (Chang and Chin, 2018). Considering the above, in our study, we focused the attention on the information.

A comparative study between the best place to work and charities was performed, focusing the attention and quantifying the web section devoted to careers and concrete information related to goods practices affecting the workers well-being. We used the qualitative method and the content analysis for collecting data from the corporate websites in a deductive approach. Initially, the Uniform Resource Locators for the 100 Best Place to Work and 100 charities corporate websites were identified. Researchers checked if websites had a recruiting web and explored whether they published the benefits to employees. Given that 97 organizations were not providing information related to our research purpose, they were removed from the study. Once the 103 sites to be deeply analyzed were identified, a three-step coding process was followed.

First, and according to the previous literature review, the measures that favor physical well-being in the workplace were fitness and massages, cafeterias, and transport (ELP, CP, and ETP, respectively). The measures that favor the psychological well-being of the employees were EAP, recognition programs, and referral programs (denoted as EAP, ERP, and ERFP, respectively). Later, the initial four websites from the list were coded independently by each of the four coders exploring the measures directed to the physical and psychological well-being. Given the number of websites, the variety of types of organization as goals and sectors, and the need to ensure reliability between coders, this step was done as a training that would ensure that all of the authors followed a similar procedure and would filter possible misunderstandings. In this step, coders met to compare their coding and discuss when there were coding differences. Once total agreement was achieved between coders, the agreed coding was used for the rest of the websites. As the last step for coding, the best and the charities websites were randomly divided between the coders. Data were collected in the form of individual actions related to well-being, in which the emphasis was placed on an in-depth understanding and description of the actions found and not only on determining frequencies.

## Results and Discussion

Data gathered served for a twofold purpose. First, it is a descriptive analysis to characterize both groups, best and charities, in relation to well-being practices to attract talent in their websites. Second, data served to create a weighted index for benchmarking purposes, using as weights the Millennials’ well-being preferences at work.

The description of the areas that were coded and examples of what counted in each category is shown in Table 1. Additional examples for a better comprehension are provided in Annex 1.

**TABLE 1:** Coding physical and psychological well-being.

**Area/Code**	**Description**	**Example best**	**Example charities**
Education and lifestyle (ELP)	Company assistance with expenses related to improving and maintaining employees’ state of physical fitness	Genentech. Fitness: employees have access to B34, also known as the “Hub,” our free, on-campus fitness center. Genentech employees at other locations can be reimbursed for individual membership at local health clubs	Population Services International: Love rock climbing? Or yoga? PSI will reimburse you for health and fitness expenses so you can get out and be active
Cafeteria (CP)	Assistance in the form of a smaller or larger amount or provision by the company of the cafeteria service (beverages and/or food) for employees	Salesforce. Enjoy bottomless gourmet snacks and beverages in the employee social lounges on every floor	Mayo Clinic: You can also choose from a broad variety of other benefits for Mayo Clinic employees including: Employee Cafeterias
Transport (ETP)	Assistance in the form of a smaller or larger amount or provision by the company of the transport service (commute and/or parking) for employees	Quicken Loans Parking and Transportation Keep your car safe and secure with free parking and convenient shuttle services	Mercy Corps contributes to an annual pass on public transportation. Bike storage, lockers and showers at Portland Headquarters
Assistance (EAP)	Instruments which organizations provide for their employees to deal with complicated work and family situations which have a negative impact on their quality of life	Texas Health Resources Wellness Employee Assistance Program: All employees can utilize our voluntary, confidential program. You and your dependents have access to unlimited telephone counseling and up to six face-to-face visits with a counselor per issue, per year	World Wildlife Fund Employee Assistance Program: Provides confidential short-term counseling services for employees and their families in a variety of areas including stress management; legal or financial issues; alcohol and drug abuse; and information on elder care, family, and education resources
Recognition (ERP)	Programs designed for the organization to explicitly show interest in and appreciation for good workers	Navy Federal Credit Union Recognizing Excellence We are proud to acknowledge our employees for their contributions to our organization by including award for: Superior performance Years of service Contributions on projects of impact Generating new ideas to improve our organization	JDRF International Values our staff members. That is why we offer competitive salaries and generous benefits. Include generous paid time off, (…) and recognition and tenure programs
Referrals (ERFP)	Programs designed for the organization to positively value employees who have suggested candidates to cover openings in the company who have subsequently turned out to be suitable for the post	Camden Property Trust. Employee Referral Program. Camden appreciates employees who do a great job referring people who share our values and commitment to be the best. As a thank you, employees are eligible for a referral bonus after the new hire’s first 6 months with Camden	Feeding America staff have the opportunity to help bring new talent into vacancies through our employee referral program. Successful referrals result in a taxable bonus

Descriptive statistics and mean difference test are reported in Table 2. Overall, the present sample is only ∼50% of the initial sample of 200 organizations. Concretely, only 44% of charities and 59% of companies had information on their webpages related to work well-being as a tool for attracting and retaining talent. The difference between means for the groups charities–best was significant for three aspects of well-being (ELP = 2.316, *p* < 0.001; CP = 2.06, *p* < 0.001; ETP = 3.135, *p* < 0.001). Organizations considered the best place to work showed higher means for ELP and CP. On the contrary, ETP showed higher mean in charities.

**TABLE 2:** Descriptive results.

	**Charities**			**Best**			**Mean difference test**		**Total**		
	**Sum (*N* = 44)**	**Mean (standard error) (*N* = 44)**	**%**	**Sum (*N* = 59)**	**Mean (standard error) (*N* = 59)**	**%**	**Mean difference**	***t* value (*p*)**	**Sum (*N* = 103)**	**Mean (standard error) (*N* = 103)**	**%**
ELP	12	**0.27 (0.451)**	27	29	**0.49 (0.504)**	49	0.219	**2.316^∗^ (0.000)**	41	0.40 (0.492)	40
CP	3	**0.07 (0.255)**	7	12	**0.20 (0.406)**	20	0.135	**2.06^∗^ (0.000)**	15	0.15 (0.354)	15
ETP	19	**0.43 (0.501)**	43	9	**0.15 (0.363)**	15	−0.279	**3.135^∗^ (0.000)**	28	0.27 (0.447)	27
EAP	19	**0.43 (0.501)**	43	25	**0.42 (0.498)**	42	−0.008	−0.08 (0.872)	44	0.43 (0.497)	43
ERP	4	**0.09 (0.291)**	9	10	**0.17 (0.378)**	17	0.079	1.192 (0.019)	14	0.14 (0.344)	14
ERFP	3	**0.07 (0.255)**	7	3	**0.05 (0.222)**	5	−0.017	−0.361 (0.463)	6	0.06 (0.235)	6
*N* valid	**44**			**59**					**103**		

*Values in bold show significant difference.*

Given the different aspects included in well-being programs, and the fact that not all of them will be equally appreciated for job seekers, a weighted index was created in order to compare the organizations:

$$I\,n\,d\,e\,x=\sum_{i=1}^{n}B_{i}\,x\,V_{i}$$

where *B*_*i*_ is the benefit offered, and *V*_*i*_ is the weight provided by jobseekers. In our study, we considered Millennials, the cohort with birth years ranging from the early 1980s to 2003, as the emerging work force at the moment (Payment, 2008; Sandeen, 2008). In fact, the first Millennial college graduates entered the workforce around 2004, and they will continue entering the labor market until 2022 (Hershatter and Epstein, 2010). Their distinctive relationship with technology has been recognized, and, consequently, it is relevant for the purpose of this study to know their preferences regarding well-being programs as potential jobseekers online.

To calculate the index, we developed an *ad hoc* self-administered questionnaire in Google Forms for a convenience sample of Millennials conformed by finalist students at the university to which the authors of this study belong. The questionnaire distributed to students included the six factors considered in this study – ELP, CP, ETP, EAP, ERP, and ERFP – with a simple explanation and several examples for guaranteeing the student’s comprehension. A 5-point Likert scale of importance was used, where 1 was “not very important for me” and 5 was “very important for me” when choosing my future job. The sample was selected considering the finalist students closest to their first job seek. To ensure diversity in profiles and the non-existence of biases in the convenience sample, previously, we instructed the participant students about well-being at work, explaining also the meaning of different kinds of benefits.

A total of 131 finalist completed the survey. The average age of the participants was 20 years, 42% men and 58% women. Once data were collected and analyzed, for an easier interpretation, the index was standardized (ZIndex). Thus, a score of 0 would indicate that the organization is in the average, the positive values would indicate a level of well-being better than the average, and negative values would indicate that these companies are below the standard. Finally, the ranking of organizations that have achieved a better score in the index is shown in Table 3.

**TABLE 3:** Top 10 index well-being ranking.

**Group^∗^**	**Position in the original rank**	**Organization**	**ZIndex**	**Category**
1	64	World Wildlife Fund	2.3267	Environment/Animal
0	41	Scripps Health	2.0956	Health care
1	14	Goodwill Industries International	2.0648	Domestic needs
1	61	Combined Jewish Philanthropies	2.0648	Domestic needs
1	73	JDRF International	1.5581	Health care
0	36	David Weekley Homes	1.3903	Construction
0	42	Navy Federal Credit Union	1.3903	Financial services
1	44	Memorial Sloan Kettering Cancer Center	1.3595	Health care
1	80	Smithsonian Institution	1.3595	Travel & leisure
0	50	Burns & Mcdonnell	1.3595	Professional services

*^∗^, charities; 0, best companies to work for.*

The findings of the study showed that the organizations of the sample, although they are considered top organizations – the largest charities and the best places to work for – still reported moderate levels of work well-being information in their webpages. Given the importance of well-being in OR for attracting talent, this aspect should be improved in both groups.

Related to the different programs to promote well-being at work, statistically significant differences exclusively occur in tools related to physical well-being and not in programs devoted to psychological well-being. It should be noted that both the promotion of physical activity and coffee shops (ELP and CP, respectively) have a significantly higher use in the Best than in the Charities group. However, the tools to facilitate commute, travel, or provision by the organization of the transport service to employees (ETP) is significantly better considered in Charities. Some interesting examples come from the Best group, such as an innovative travel solution for breastfeeding moms at SAP America INC: “Milkstork is a breast milk delivery service for business traveling moms. Nursing moms can use overnight express shipping or easy toting of breast milk home to baby when they are on the road.”

The results also show that the position of each one in their respective rankings, Best or Charity, is not related to being champion in promoting well-being at work in their websites. According to the performed content analysis, and the subsequent Millennials’ opinion, undoubtedly, the winner of the ranking was WWF, which was placed over the average, in number 64, in the charities list. It is also remarkable that a charity is on the top, ahead of for-profit companies, considered the best places to work for. This reflects the potential of any non-profit organization in attracting talent through OR by disseminating well-being at work online. Statements such as, “At WWF, we work every day to make sure our world is and will continue to be a healthy and positive place to live in” or “We see the workplace as one small part of that greater world, and so it’s very important to us that our work environment is equally healthy and positive,” are good examples for attracting talent online, by showing their employee well-being orientation in the webpage. Even the support that WWF gives to its employees goes ahead their work life, as it is shown in this last statement, “We strive to give our employees the kinds of benefits they need to support them in their work and home lives.”

**FIGURE 1:**
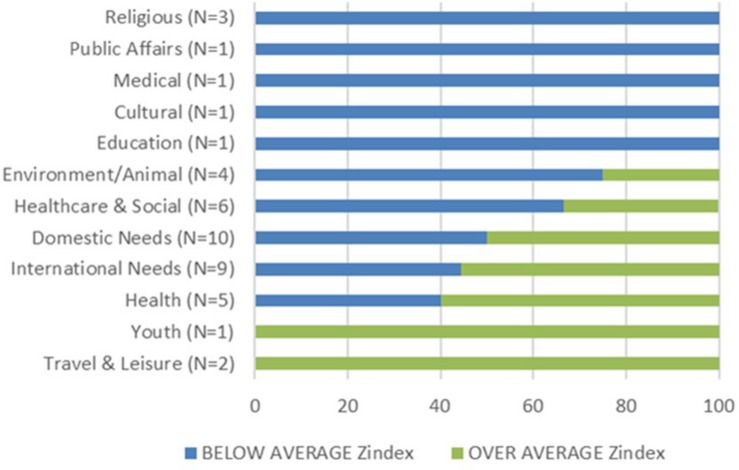
Charities over and below average Zindex.

Considering most preferred well-being programs, our ranking shows in which sectors charities were stronger: health, social, and domestic needs. However, and considering that the analyzed charities belong to 12 different sectors, Figure 1 shows how the representation of five of them was totally below the average. We want to highlight that the religious sector was one of them. Only two sectors were located totally over the average: youth and travel and leisure. The case of the environment/animal sector is remarkable, where only one organization is above the average in the index: the WWF, which is also the organization that, from all the analyzed organizations (whether charities or not), achieved the best positioning.

## Conclusion

Given the lack of research on HRM on the voluntary sector, this study sheds light on what could be learned by charities from the best in managing talent in the for-profit sector. We have done so by observing what they were reporting on their webpages, with special attention to the well-being practices whereby organizations become employers of choice. Despite the voluntary organizations’ adoption of technology and social media, the research shows that their networking site purposes and HRM efforts are still not directed toward OR.

According to the results obtained in comparing best places to work and charities, we can say that the best places to work are the most advanced in the topic under study considering the number of organizations showing well-being programs as part of their OR strategy. No clear differences have been found between the two groups with regard to psychological well-being programs among employees, but charities must improve with regard physical well-being. Although ETP is better considered in charities, ELP, and CP are more developed in the best places to work. Charities could reinforce these programs because they can give opportunities for growth on the job, allowing employees capabilities development and their growth as people (McCauley and Wakefield, 2006). We can appreciate the role that coffee shops play to make the employee life easier, facilitating both their reconciliation with personal life and their socialization within the company.

The employee referral programs is still underused in both groups and must be reinforce. The ERFP programs, when fair, competitive, equitable, and recognize the value of employee contributions, make the organization more attractive. The ERFP that can allow charities having meaningful social interactions and form strong networks at the workplace that last beyond the current place of employment also makes for a more attractive organization (Powell, 2009).

One charity has revealed as well-being champion in the study; it was WWF. Charities must reinforce their websites to attract talent as part of their OR strategies, and benchmarking efforts could be helpful in increasing the charities’ attractiveness in the labor market in the near future. It will be essential, in the OR process of charities, to understand the relevance of their web content because that will affect the intentions of potential applicants. Thus, assuming that the best employees want to work for the best organizations, charities needed an excellent employer image and reputation to be successful in the war of talent, which is not possible without being recognized for delivering quality services, behaving ethically, and doing a good HRM (Edwards, 2009). In this sense, in line with Pandita and Ray (2018), we have shown that well-being at work is a good point to make charity more appealing for Millennial jobseekers online.

To conclude, this work contributes to the academic literature on the future of work on voluntary sector in different ways. First, our findings are consistent with the research defending the role of OR in the war for talent (Ashton and Morton, 2005; Manyika et al., 2015; Bostjancic and Slana, 2018) and those suggesting the need for professionalization of HRM to increase efficiency (Nickson et al., 2008; Ridder et al., 2012). Our second contribution is for practitioners because some practical implications emerge from the study. Our findings can be used by researchers and HR managers in charities or external professional services providing support to them to attract and retain the best human capital in the near future. Benchmarking efforts can be helpful to increase the charities’ attractiveness in the labor market starting from examining the work conditions of workers in non-lucrative organizations and designing improvement programs following the example of the well-being champions.

Acknowledging internet and webpages as a major communications medium welcomed by content analysis researchers (Weare and Lin, 2000), the present study has some limitations. Reliability and validity of any content analytic research must be born in mind because the study is cross-sectional, based on secondary information self-reported by the organizations of the sample in their webpages. Complementing well-being at work as a strategic issue for charities, we highlight the need to study work-life balance programs to help charities to attract and retain talent (Cappelli, 2000; Hill et al., 2008; Allen T.D. et al., 2013). The findings of the present study can be extended to future comparative analyses in other non-profit organizations. In addition, other HRM practices are susceptible to be explored for OR purposes apart from the well-being such as, for instance, work–life balance programs.

## Data Availability Statement

The datasets generated for this study are available on request to the corresponding author.

## Ethics Statement

Ethical review and approval was not required for the study on human participants in accordance with the local legislation and institutional requirements. The patients/participants provided their written informed consent to participate in this study.

## Author Contributions

All authors listed have made a substantial, direct, and intellectual contribution to the work and approved it for publication.

## Conflict of Interest

The authors declare that the research was conducted in the absence of any commercial or financial relationships that could be construed as a potential conflict of interest.

## annexture

**ANNEX 1:** Quotations about physical and psychological well-being.

**Education and lifestyle (ELP)**
∙ Salesforce (Best): “Relax your way with stress relief via our meditation rooms”
∙ UJA/Federation of New York (Charity): “(…) and for a small cost you can take part in on-site yoga and exercise classes at our Manhattan office. Wellness matters to us”
**Cafeteria (CP)**
∙ Quicken Loans (Best): “We want you to be happy, and being happy means being well-fed (in our opinion). Enjoy free cappuccinos, slushies, popcorn, and other tasty titbits all day every day in all locations”
∙ Task Force for Global Health (Charity): “All Task Force employees are Emory University employees which includes a competitive benefits package: (…) provides our employees access to restaurants, coffee shops (…) within walking distance of our campus”
**Transport (ETP)**
∙ Genentech (Best): “gRide, Genentech’s employee transportation program, helps you get to and from work stress-free. Save time and money while supporting the environment”
∙ Smithsonian Institution (Charity): Pretax Commuter Benefit Program/Transit Subsidy Program: “The Smithsonian Transit Subsidy Program provides up to the current maximum per month for mass transit costs incurred while commuting to and from an employee’s work duty station. The Pretax Parking Program allows employees to use Pretax dollars to pay for eligible parking expenses while at work. The Commuter Bicycle Reimbursement Program provides employees with reimbursement for reasonable expenses incurred for the purchase of a bicycle or bicycle maintenance, repair, and storage if the bicycle is regularly used for travel between the employee’s home and place of employment”
**Assistance (EAP)**
∙ Capital One Financial Corporation (Best): “Capital One’s Employee Assistance Program offers face-to-face or telephone counseling and provider referrals. The program also provides information and services for a wide range of needs, such as moving, coping with change or even finding a pet sitter”
∙ Feeding America (Charity): “Feeding America has counseling professionals available to assist employees and their families with work and life issues. The program is designed to provide immediate confidential counseling services and referrals when necessary”
**Recognition (ERP)**
∙ Camden Property Trust (Best): “An Apartment Community Excellence (ACE) cares about costumers and colleagues so much they go beyond simply doing the right thing. We love celebrating people like that. Employees who exemplify Camden’s values are honored at the annual ACE Awards banquet, hosted by CEO wearing a Camden maintenance shirt to show his appreciation for frontline employees”
∙ Compassion International (Charity): Performance and recognition program “We actively support and intentionally recognize the achievements of our team.” Milestone Recognition, Objective Development and Monitoring. Anniversary travel options:
–5 years: Central America and the Caribbean
–10 years: South America
–15 years: Africa
–20 years: Asia
**Referrals (ERFP)**
∙ Baird (Best): “Additional Benefits: (…) Employee Referral Bonus”
∙ Combined Jewish Philanthropies (Charity): “Other Benefits: (…) $500 employee referral bonus”
